# Visual snow syndrome after start of citalopram—novel insights into underlying pathophysiology

**DOI:** 10.1007/s00228-020-02996-9

**Published:** 2020-09-16

**Authors:** Ozan E. Eren, Florian Schöberl, Christoph J. Schankin, Andreas Straube

**Affiliations:** 1 Department of Neurology, LMU Munich, University Hospital-Großhadern, Marchioninistr. 15, 81377 Munich, Germany; 2 Department of Neurology, Inselspital, Bern University Hospital, University of Bern, Bern, Switzerland

Dear Sirs,

Patients with visual snow (VS) describe tiny flickering dots in the entire visual field often compared with the TV static in case of a badly adjusted analog signal. It can be episodic with migraine [1], but is debilitating when continuously present. When occurring together with visual (palinopsia, enhanced entoptic phenomena, photophobia, and nyctalopia) and a variety of non-visual symptoms (tinnitus, concentration loss, etc.), one speaks of visual snow syndrome (VSS) [2].

Pathophysiological mechanisms are still not well-understood, and pharmacological treatment options are sparse. We present a case of VSS onset after citalopram intake and discuss this constellation in the light of “Hallucinogen Persisting Perception Disorder” (HPPD) with possible shared receptor mechanisms.

## Case report

Two weeks after starting with citalopram 20 mg to treat postpartum depression, a 31-year-old female developed visual disturbances in terms of white and black dots in the entire visual field, intermittent bright flashes, entoptic phenomena, swirls, photophobia, and additionally increased irritability to external sensory cues. Past medical history was unremarkable except for episodic migraine without aura (every 8 weeks; MIDAS 0p). There was moderate anxiety and depression as assessed by the GAD-7 anxiety scale (7p) and the PHQ-9 depression scale (10p) at that time. There was no use of hallucinogenic substances in the past. Diagnostics including thorough clinical neurological and ophthalmological examination, visual evoked potentials (16 k and 64 k), and MRI-scan of the brain were unremarkable. VSS persisted after stopping citalopram. An additionally used causality assessment tool (CAT) for adverse drug reaction (ADR), namely the Liverpool ADR CAT, indicated a possible adverse drug reaction [3].

## Case discussion

Individual symptoms of VSS can overlap with conditions such as visual migraine aura [2, 4] and HPPD [5, 6]. Particularly, VSS and HPPD symptoms largely overlap and therefore might share common pathophysiological mechanisms in terms of involved cerebral networks and changes in synaptic transmission. The drug lysergic acid diethylamide (LSD) mediates its hallucinogenic effects mainly via activating the serotonergic 5HT_2a_-receptor [7]. Even one-time consumption of LSD can cause persistent HPPD with VS-like symptoms [6]. Interestingly, there are reports on visual sensations arising years after LSD abuse triggered by the intake of selective serotonin reuptake inhibitors (SSRI) in patients with former HPPD [7]. Regarding our case, the hypothesis arises that changes in serotonergic synaptic transmission, particularly via 5HT_2a_-receptors, in primary and/or secondary visual cortical areas might underlie VSS as well as HPPD. Functional imaging studies revealed increased metabolism in secondary visual cortical areas such as the lingual gyrus in VSS [4]. There is evidence for a particularly high density of 5HT_2_-receptors of projections from the lateral geniculate body of the thalamus to the primary visual cortex [8, 9]. It is hypothesized that a shift in the balance of excitatory and inhibitory synaptic transmission in the visual cortex towards more excitation due to a decrease in either the number or the functional activation of inhibitory serotonergic interneurons might play a crucial role in HPPD after LSD abuse [6]. Thus, in subjects with an increased vulnerability, VSS might also be triggered by minor changes in serotonergic transmission due to SSRI, like in our case. Further evidence for the important role of serotonergic transmission derives from the at least partial effectiveness of lamotrigine in both conditions [10–12], as lamotrigine also has an inhibitory function on 5HT_2a_-receptors [13].

## Conclusion

This case report gives further insight for a possible role of changes in serotonergic transmission in brain regions important for visual processing as potentially underlying pathophysiological mechanism of VSS. Furthermore, common pathophysiological changes in VSS and HPPD on the level of cerebral network activations and synaptic transmission seem plausible.

## Data Availability

The datasets used and/or analyzed during the current study are available from the corresponding author on reasonable request.
